# 2088. Real-World Geographic Variations of HIV Diagnosis Rates among Individuals Prescribed and not Prescribed Oral HIV Pre-Exposure Prophylaxis (PrEP) Regimens in the United States

**DOI:** 10.1093/ofid/ofac492.1710

**Published:** 2022-12-15

**Authors:** Li Tao, Jen Thorburn, Amanda Kong, Debra Irwin, Christoph C Carter, Moupali Das, Julie Paone

**Affiliations:** Gilead Sciences, Foster City, California; Aetion, New York, New York; Aetion, New York, New York; Aetion, New York, New York; Gilead Sciences, Foster City, California; Gilead Sciences, Foster City, California; Aetion, New York, New York

## Abstract

**Background:**

Declines in the diagnoses of new HIV infections have been reported in geographic areas with higher uptake of PrEP among persons who would benefit from PrEP (PWBP). In the present analysis, we examined the rate of new HIV diagnoses in PWBP prescribed PrEP and those not prescribed PrEP in order to understand the current and potential impact of PrEP in differing geographies.

**Methods:**

We estimated the rate of new HIV infections among PWBP not prescribed PrEP with a previously described model (Mera *et al.* 2019) using a combination of published reports and a claims database. HIV rates for individuals prescribed F/TAF or F/TDF (including brand and generic) for PrEP between 10/3/2019 (F/TAF approval) and 6/30/2021 were analyzed from claims data. For those prescribed PrEP, new infections included new HIV diagnosis or addition of HIV treatment within 10 days of PrEP discontinuation.

**Results:**

Overall, the US HIV diagnosis rate was 61% lower among PWBP prescribed PrEP (1.33 per 100-person year [95%CI: 1.24 – 1.42]) compared to those not prescribed PrEP (3.38 [3.35 – 3.42]). New HIV diagnosis rates by state among PWBP not prescribed PrEP ranged from 0.41 (0.22 – 0.78) in Vermont to 9.96 (9.53 – 11.54) in Mississippi (Figure). The Southern US had the highest regional HIV diagnosis rate among PWBP not prescribed PrEP (4.20 [4.14 - 4.26], Table). HIV diagnosis rates by state among PWBP prescribed PrEP were lower, ranging from 0 to 2.40 (0.05 – 4.75). Eleven states had a greater than 85% lower HIV diagnosis rate among PWBP prescribed PrEP versus those who were not prescribed PrEP, and the greatest differences in diagnosis rates by PrEP prescription status were observed in the Southeastern US states.

**Figure:**
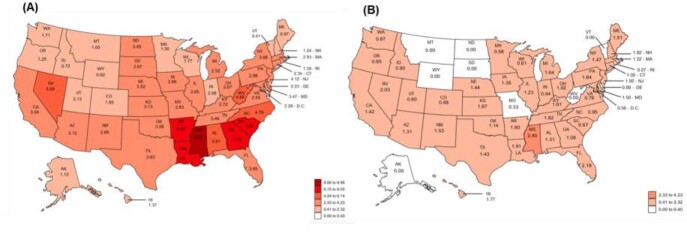
The estimated rates of new HIV diagnosis (per 100 person-years) among PWBP who were not prescribed PrEP in 2019 (A) and those prescribed PrEP from 10/3/2019 to 6/30/2021 (B) in the United States.

**Table:**
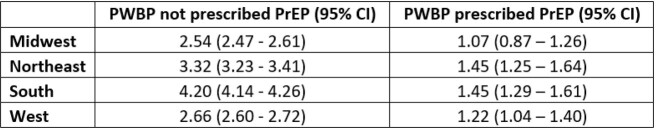
Regional variations of new HIV diagnosis rates among PWBP who were not prescribed PrEP in 2019 and those prescribed PrEP from 10/3/2019 to 6/30/2021 in the United States.

**Conclusion:**

This study suggests that substantial reductions in HIV diagnosis rates have occurred in people prescribed PrEP in the US. We found large geographic variations in HIV diagnosis rates, with the largest differences by PrEP prescription status occurring in the Southeast US, underscoring the need for PrEP expansion and its potential impact on the HIV epidemic in that region. Furthermore, our findings demonstrate an approach of estimating HIV rates for PWBP prescribed and those not prescribed PrEP, which may be useful in supporting targeted delivery of HIV prevention services in the US.

**Disclosures:**

**Li Tao, MD, PhD**, Gilead Sciences: Employee|Gilead Sciences: Employee|Gilead Sciences: Stocks/Bonds|Gilead Sciences: Stocks/Bonds **Jen Thorburn, n/a**, Gilead Sciences: Grant/Research Support **Amanda Kong, DrPH**, Gilead Sciences: Grant/Research Support **Debra Irwin, PhD**, Gilead Sciences: Grant/Research Support **Christoph C. Carter, MD, PhD**, Gilead Sciences: Employee|Gilead Sciences: Employee|Gilead Sciences: Stocks/Bonds|Gilead Sciences: Stocks/Bonds **Moupali Das, MD**, Gilead Sciences: Employee|Gilead Sciences: Employee|Gilead Sciences: Stocks/Bonds|Gilead Sciences: Stocks/Bonds **Julie Paone, MPH**, Gilead Sciences: Grant/Research Support.

